# Atypical Oral Presentation of Giant Cell Arteritis With Subsequent Middle Cerebral Artery Involvement

**DOI:** 10.7759/cureus.99107

**Published:** 2025-12-13

**Authors:** Alamin Alkundi, Christa Mathew

**Affiliations:** 1 Medical Education, Kent and Medway Medical School, Canterbury, GBR; 2 Diabetes and Endocrinology, Liverpool University Hospitals, Liverpool, GBR; 3 General Internal Medicine, North Devon District Hospital, Barnstaple, GBR

**Keywords:** anterior circulation stroke, diagnostic and therapeutic challenge, giant cell arteritis prognosis, vascular dysphagia, vasculitis mouth ulcer

## Abstract

Giant cell arteritis (GCA) is a large-vessel vasculitis classically affecting the temporal arteries and associated with visual loss and constitutional symptoms. We report a rare and diagnostically challenging case of GCA in an elderly patient presenting with progressive visual impairment, anterior circulation stroke, vasculitic mouth ulcer, and dysphagia requiring nasogastric feeding. Diagnosis was confirmed by ultrasound findings without histological confirmation. This case underlines the potential for GCA to present with multisystem ischemic manifestations and the importance of early recognition and corticosteroid therapy.

## Introduction

Giant cell arteritis (GCA), also known as Horton’s disease - a large‑vessel vasculitis characterized by granulomatous inflammation of medium and large-sized arteries - is the most common primary systemic vasculitis in adults over the age of 50 [1]. It shows a strong predilection for women, particularly those of Northern European ancestry. The classical presentation includes new‑onset headache, scalp tenderness, jaw claudication and visual disturbance, though the clinical spectrum is heterogeneous and may overlap with other conditions common in older adults [2,3]. Delayed diagnosis of giant cell arteritis can lead to catastrophic outcomes, including irreversible vision loss and cerebrovascular complications, highlighting the critical importance of early recognition and prompt initiation of high‑dose corticosteroid therapy. Despite improvements in vascular imaging and the introduction of targeted biologic therapies, delays in the diagnosis of giant cell arteritis remain frequent, particularly when initial symptoms are nonspecific or mimic other conditions [4]. We present the case of an elderly woman with coexistent polymyalgia rheumatica and an autoimmune comorbidity who developed bilateral blindness, dysphagia and functional decline due to delayed recognition of GCA, illustrating the severe morbidity that may result when clinical suspicion is not immediately raised [5].

## Case presentation

**Figure 1:**
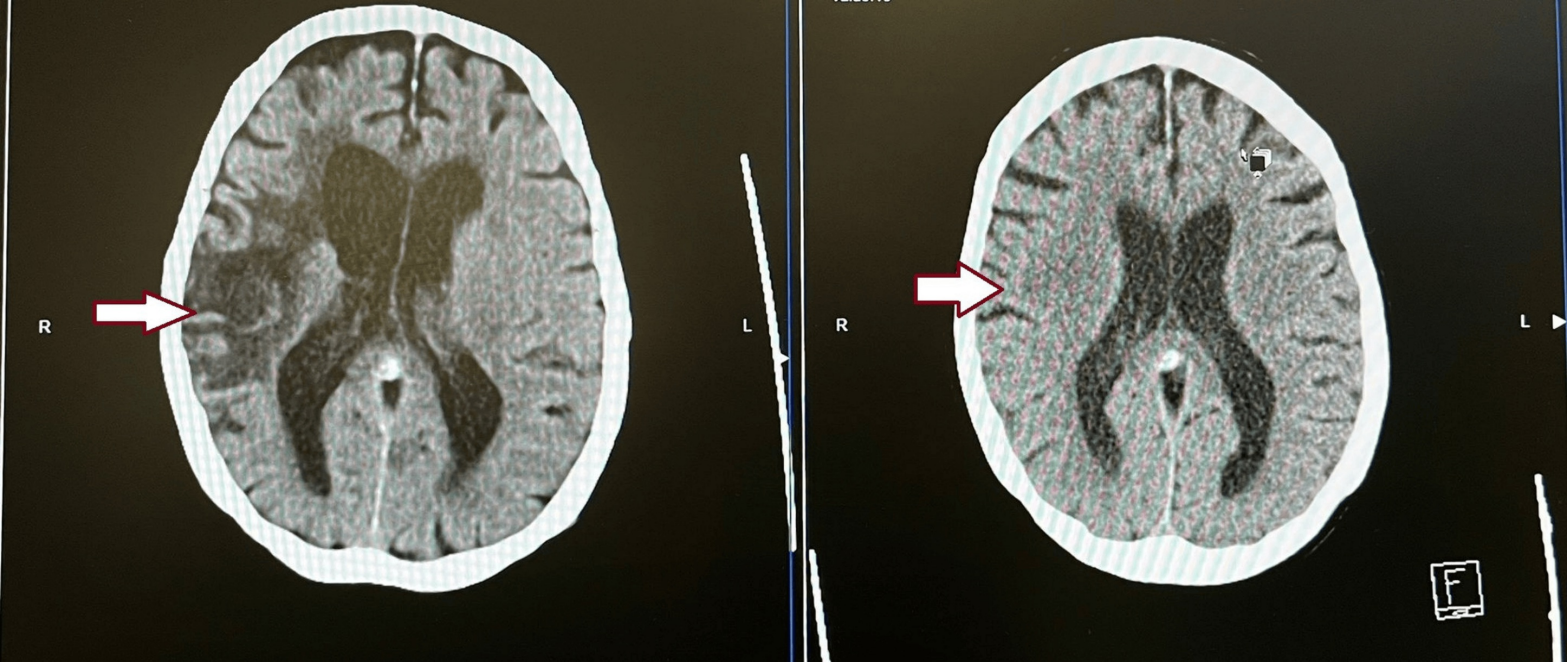
(1a) (2025) well established (chronic) right middle cerebral artery infarction with encephalomalacia which has significantly advanced and new since 2020 CT findings; (1b) (2020) low attenuation affecting grey and white matter in the right frontal lobe in part of the right middle cerebral artery; consistent with acute infarct Arrow points out the extension of infarct from the right frontal region in 2020 to the right temporal, insula and basal nuclei region with brain tissue swelling in 2025.

An 87-year-old woman with a background of autoimmune hemolytic anemia on long-term low-dose prednisolone, polymyalgia rheumatica, hypertension, peripheral vascular disease, and a prior anterior circulation stroke presented with sudden visual loss, right temporal headache, and progressive dysphagia of two weeks' duration. Two weeks earlier, she had been reviewed for persistent right mandibular pain in the context of poor oral hygiene by a dentist and was diagnosed with a gingival abscess and multiple carious teeth, and completed a 5-day course of amoxicillin. She subsequently developed worsening right-sided headaches, progressive visual blurring and swallowing difficulties. She was admitted following this presentation, as there was progressive concentric visual field loss and frequent collisions with objects. Ophthalmological evaluation demonstrated a retrospective giant cell arteritis probability score of 10, anterior ischemic optic neuropathy of the left eye consistent with artery origin, and right optic atrophy, likely from a previous ischemic insult. The inflammatory markers were elevated, as shown in Table 1. She was commenced on intravenous methylprednisolone (1 g daily), with rapid resolution of headache and jaw pain and partial recovery of light perception in the left eye. After a 5-day course, she was discharged on a tapering oral prednisolone regimen, with rheumatology follow-up and planned temporal artery biopsy/ultrasound for diagnostic confirmation. After two days, on this current admission, she awoke with complete visual loss, severe band-like headache, worsening dysphagia, and functional decline. The headache was more over the right side, constant, sharp in character, radiating to the right eye, rated 8/10 in severity, and refractory to analgesics. Examination revealed cachexia, right eye blindness with residual light perception in the left eye, flaccid dysarthria involving palatal and lingual phonation, wet upper airway cough with hypoxemia requiring supplemental oxygen, and a thickened, pulseless temporal artery. Neurological examination revealed no acute lateralising signs. Differential diagnoses included oromandibular claudication, progression of giant cell arteritis, and vasculitis stroke. Laboratory investigations showed elevated inflammatory markers and metabolic alkalosis with hypokalemia. CT brain demonstrated extension of her previous middle cerebral artery infarct with encephalomalacia as seen in Figure 1a-1b.

**Table 1:** Laboratory findings

Investigations	Week 1	Week 2	Week 3	Week 4	Normal range
C-Reactive Protein	78 mg/L	61 mg/L	31 mg/L	73 mg/L	<=5 mg/L
White blood cells	9.9 × 10⁹/L	14.9 × 10⁹/L	11.8 × 10⁹/L	21.3 × 10⁹/L	3.6-11.0 × 10⁹/L
Neutrophils	7.58 × 10⁹/L	12.09 × 10⁹/L	10.40 × 10⁹/L	17.38 × 10⁹/L	1.8-7.5 × 10⁹/L
Potassium	3.5 mmol/L	3.0 mmol/L	4.1 mmol/L	3.3 mmol/L	3.5-5.1 mmol/L
pH	-	7.488	-	7.489	7.32-7.43
Bicarbonate	-	34.4 mmol/L	-	33.2 mmol/L	22-29 mmol/L

**Figure 2:**
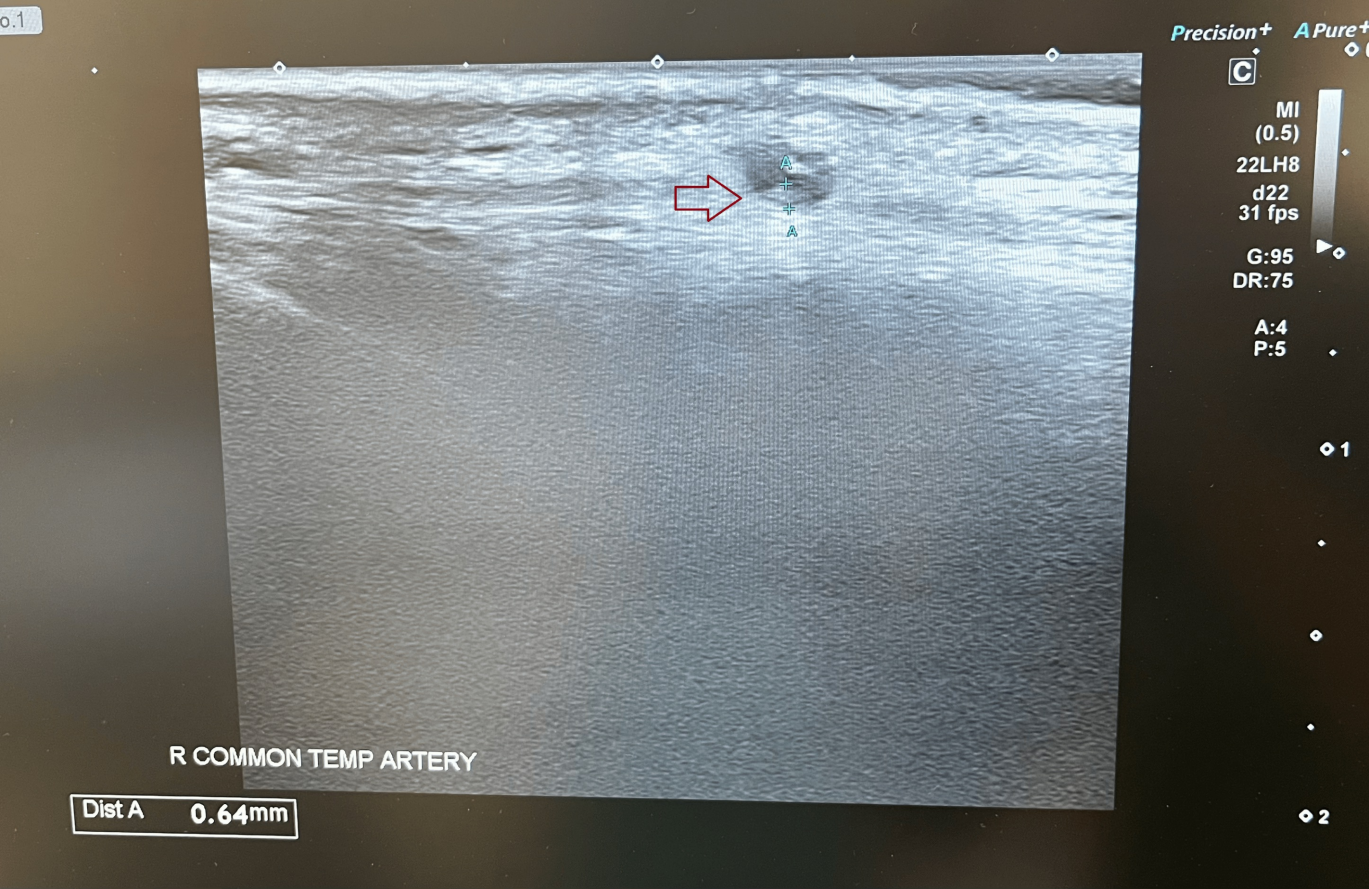
Temporal artery ultrasound showing intima thickness of 0.64 mm and minimal blood flow through it. Arrow points out the temporal artery blood vessel with an abnormal thickness >0.3 mm, indicative of vasculitis, also known as halo sign.

Temporal artery ultrasound, as seen in Figure 2, demonstrated right temporal artery intima-media thickness of 0.64 mm (reference range: 0.25-0.44 mm), reduced flow in the common temporal artery, and a positive compression sign, confirming giant cell arteritis. Maxillofacial review noted healing of the previously documented gingival abscess but poor oral hygiene. Rheumatology concluded that her deterioration was due to giant cell arteritis complicated by oral vasculitis ulceration with bacterial superinfection and extension of cerebrovascular disease. Prognosis was considered poor in the context of delayed treatment due to the variable nature of presentation, severe frailty, and poor baseline function. She was treated with intravenous methylprednisolone for 48 hours before transition to nasogastric prednisolone following swallow assessment.

Tocilizumab was considered but deemed unlikely to provide clinical benefit. In view of her profound weight loss (6 stones), frailty, and limited recovery potential, a palliative approach was adopted, with corticosteroid therapy aimed at preventing further visual and soft tissue compromise.

## Discussion

Giant cell arteritis (GCA) is the most common among the rare, primary systemic vasculitis in adults over the age of 50, with an incidence that increases with advancing age and a strong female preponderance [6]. The classic clinical features include new-onset temporal headache, scalp tenderness, jaw claudication, and visual symptoms, often accompanied by systemic manifestations such as weight loss, fatigue, and polymyalgia rheumatica [7]. This case highlights several key challenges in the diagnosis and management of GCA. Firstly, the patient initially presented with symptoms that could be attributed to alternative pathologies, including dental infection and post-stroke sequelae, which contributed to a diagnostic delay. Atypical or overlapping presentations are well recognized in GCA and may obscure timely diagnosis [8,9].

Visual loss remains the most feared complication of GCA, typically due to anterior ischaemic optic neuropathy (AION), and is often irreversible despite corticosteroid therapy [10]. Our patient had progressive visual decline culminating in bilateral blindness, reflecting both the aggressive course of arteritic involvement and the impact of delayed initiation of treatment. Prompt high-dose corticosteroid therapy is essential to prevent contralateral eye involvement and reduce systemic vascular complications [11]. The case also illustrates less commonly reported complications, including oral vasculitic ulceration with secondary bacterial infection. Oral and oromandibular manifestations of GCA are rare but have been documented, often presenting as jaw claudication or tongue ischaemia, and may be mistaken for dental pathology [12,13]. In our patient, the co-existence of a gingival abscess further complicated the diagnostic process. Neurovascular complications are another recognised manifestation of GCA, particularly large-vessel involvement leading to stroke [14]. This patient suffered extension of a previous middle cerebral artery infarct, consistent with literature reporting an increased risk of cerebrovascular events in GCA despite immunosuppressive therapy [15]. The anatomical basis is the close continuity and shared origin of the external carotid artery and internal cerebral artery at the carotid bifurcation, allowing arteritis to spread proximally and involve the internal carotid artery, with devastating effects on cerebral and ocular circulation. While corticosteroids remain the cornerstone of therapy, their long-term use is associated with significant morbidity, particularly in elderly frail patients with multiple comorbidities [16]. Tocilizumab, an IL-6 receptor inhibitor, has been shown to induce sustained remission and reduce glucocorticoid dependence in GCA [17]. However, its benefit in advanced disease with established complications is less clear. In this case, given the patient’s frailty, poor prognosis, and palliative context, the decision was made to continue steroids alone for symptom control and to prevent further devastating scalp ulceration. This case underscores the importance of early recognition and treatment of GCA to prevent irreversible complications such as blindness and stroke. It also highlights the diagnostic challenges in elderly patients with multiple comorbidities, where overlapping symptoms may lead to treatment delay. Clinicians should maintain a high index of suspicion for GCA in patients over 50 presenting with new headaches, jaw pain, or visual changes, even when alternative explanations appear plausible.

This case also highlights the importance of recognizing atypical presentations of GCA, where initial symptoms, such as jaw or oral pain and presumed dental infection, can obscure the diagnosis and delay appropriate treatment. Clinicians should maintain a high index of suspicion for GCA in older patients presenting with craniofacial symptoms, even in the absence of classical features such as temporal tenderness or visual complaints. Prompt initiation of corticosteroid therapy remains essential to prevent irreversible complications, including visual loss and cerebrovascular events, and should not be deferred while awaiting diagnostic confirmation. Furthermore, the presence of oral vasculitic ulceration and secondary infection, though uncommon, underscores the potential for widespread systemic involvement in GCA. Management in frail older adults poses additional challenges, necessitating careful consideration of treatment intensity, prognosis, and overall quality of life. A multidisciplinary and, where appropriate, palliative approach is often required to balance disease control with patient-centered care.

## Conclusions

This case underscores the catastrophic impact of delayed giant cell arteritis recognition, where diagnostic uncertainty led to bilateral blindness, cerebrovascular progression, and oral vasculitic complications. It serves as a reminder that in older adults, new-onset headache, jaw pain, or visual disturbance should immediately raise suspicion for GCA - even when a seemingly plausible alternative diagnosis exists. Swift initiation of corticosteroids remains the single most critical step in preventing irreversible disability, and a switch to steroid-sparing immunomodulating agents should also be considered in refractory cases.
